# The Results Are In: Bacterial Parasite Strives for Balance in Host Infection

**DOI:** 10.1371/journal.pbio.0040223

**Published:** 2006-05-30

**Authors:** Liza Gross

When horror-movie writers run out of ideas, they can always turn to parasites. Imagine the possibilities with flesh-eating bacteria, suicide-inducing hairworms, scalp-burrowing botflies—and castrating parasites. Such debilitating effects are an inevitable consequence of infection, but it is in the parasite's interest to avoid killing the host until it can transmit a new crop of pathogens.

In the “tradeoff hypothesis” for the evolution of virulence—how quickly a parasite kills its host—host exploitation and parasite reproduction are balanced to maximize the parasite's lifetime transmission success. But lifetime transmission success, an indicator of parasite fitness, has proven difficult to measure, leaving scant direct evidence for an optimal level of virulence.

In a new study, Knut Helge Jensen, Dieter Ebert, and colleagues worked with water fleas *(Daphnia magna)* and the castrating bacterium *Pasteuria ramosa* to investigate the relationship between parasite fitness and virulence. Castrating parasites divert resources from host reproduction toward their own reproductive ends. In the case of *P. ramosa*, that means generating transmission-stage parasites, or spores. Many castrators also boost host growth in a phenomenon called gigantism, which presumably helps support the outsized resource needs of a parasite that can account for as much as 25% of its host's body weight.

In keeping with the tradeoff hypothesis, the researchers predicted that the parasite should castrate early to optimize the appropriation of host resources, and produce intermediate levels of virulence to keep the host alive long enough to maximize spore production. They used a castrating parasite that produces copious quantities of spores but doesn't release them until the host dies, so they could estimate lifetime parasite reproduction and relate it to virulence. They found significant variation in parasite virulence, and present direct evidence linking parasite reproductive success to an optimal level of virulence, with transmission success peaking at an intermediate level of virulence.

To determine the relationship between virulence and lifetime production of transmission-stage parasites, the researchers exposed a *Daphnia* clone to bacterial spores and tracked individual host mortality. Infected *Daphnia* sustained far more casualties than either unexposed controls or exposed but uninfected individuals, with deaths beginning at 23 days old and ending at 74 days old. (The first control died at 96 days old.) Early host death (high virulence) was bad news for *P. ramosa*, since the parasite needs several weeks to produce spores. But it also didn't fare terribly well with a long-lived host (low virulence), suggesting that the bacteria in these hosts grew too slowly to reach the optimal time of killing. The highest spore production was detected in *Daphnia* expiring at middle age, likely reflecting the benefits of using host resources for spore production—which can be impressive. One clutch of host eggs corresponds to an estimated 4.5 million *P. ramosa* spores, according to a recent study. Because total spore production can be used as a proxy for parasite fitness, the researchers concluded that maximum parasite fitness derives from an intermediate level of virulence.

To determine whether genetic variation among parasite lines correlated with observed differences in virulence, the researchers infected hosts with spores collected from early dying and late-dying *Daphnia*. They found that spores from the early killing infections did indeed produce significantly higher death rates than did the late-killing spores. These results indicate that genetic variation in the parasite can influence variation in virulence, and thus affect the evolution of virulence. The correlation between an optimal level of virulence and parasite fitness may result from the strong tradeoff between host and parasite fitness, the researchers explain, which emerges as these adversaries battle for the resources needed for reproduction.

This experimental evidence for the long-held assumption that parasite fitness directly relates to virulence has important implications for a wide range of virulence-related phenomena. Estimating likely changes in parasite virulence is essential for formulating public health strategies to contain emerging parasitic diseases and for developing effective drugs and vaccines. In future studies, the researchers plan to investigate how selection pressures, such as those caused by drugs, influence parasite fitness and cause changes in virulence.

## 

**Figure:**
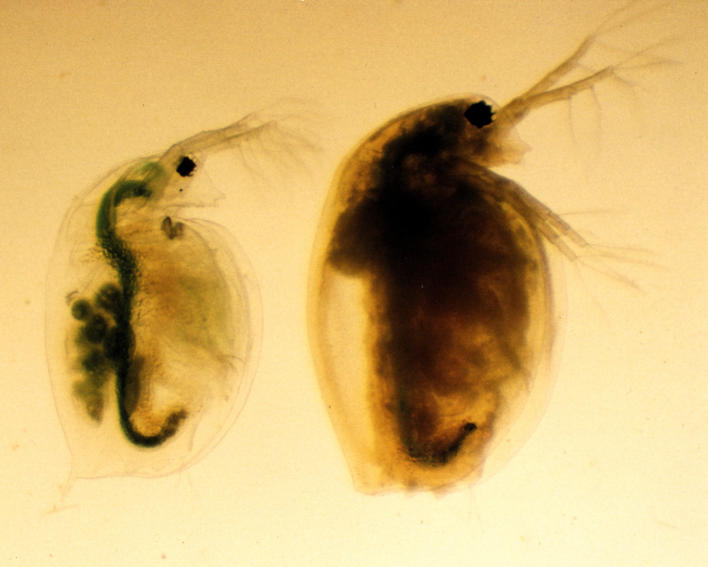
Exposing Daphnia host clones to spores from the castrating bacterium Pasteuria ramosa provides experimental evidence that parasite fitness is maximized at intermediate levels of virulence.

